# Severe Bupropion Overdose Resulting in Cardiac Arrest, Delayed Rhabdomyolysis, and Persistent Neurological Sequelae in an Adolescent

**DOI:** 10.3390/life15121918

**Published:** 2025-12-15

**Authors:** Che-Pei Chang, Po-Chen Lin, Giou-Teng Yiang, Meng-Yu Wu, Shi-Bing Wong

**Affiliations:** 1 Department of Emergency Medicine, Taipei Tzu Chi Hospital, Buddhist Tzu Chi Medical Foundation, New Taipei City 23142, Taiwan; jackchang4444@gmail.com (C.-P.C.); taipeitzuchier@gmail.com (P.-C.L.); gtyiang@gmail.com (G.-T.Y.); skyshangrila@gmail.com (M.-Y.W.); 2 School of Medicine, Tzu Chi University, Hualien 97004, Taiwan; 3 Graduate Institute of Injury Prevention and Control, Taipei Medical University, Taipei 110301, Taiwan; 4 Department of Pediatrics, Taipei Tzu Chi Hospital, Tzu Chi Medical Foundation, New Taipei City 23142, Taiwan

**Keywords:** bupropion, overdose, seizures, cardiac arrest

## Abstract

Bupropion overdose can result in severe neurological and cardiovascular toxicity. We describe a 16-year-old girl who ingested 4.2 g of extended-release bupropion (90.3 mg/kg), presenting with seizures and out-of-hospital cardiac arrest. After 21 min of cardiopulmonary resuscitation, she was resuscitated and admitted with profound metabolic acidosis and electrocardiographic abnormalities. Serum testing confirmed markedly elevated bupropion levels. During hospitalization, she developed delayed rhabdomyolysis, hypoxic encephalopathy, and persistent neurological sequelae, including Parkinsonism and cognitive deficits. Supportive care led to gradual recovery, with normalization of cardiac conduction and drug clearance by day 20, though residual deficits remained at discharge after seven weeks. This case highlights the life-threatening complications of bupropion toxicity, the delayed risk of seizures, and the need for vigilance for secondary complications such as rhabdomyolysis.

## 1. Introduction

Bupropion is an atypical antidepressant commonly prescribed for major depressive disorder, seasonal affective disorder, and smoking cessation. It primarily acts as a norepinephrine and dopamine reuptake inhibitor, with minimal serotonergic activity, and is structurally related to amphetamines. Available in immediate-, sustained-, and extended-release formulations, bupropion has a relatively large volume of distribution and is extensively metabolized in the liver via CYP2D6. Pediatric and adolescent bupropion overdose can be life-threatening, particularly when ingestion exceeds 48 mg/kg. The toxic effects, largely due to its sympathomimetic properties, include agitation, seizures, tachycardia, hypertension, and cardiotoxicity, such as QRS and QT interval prolongation.

Detailed pediatric case reports focusing on the progression from seizure to cardiac arrest, subsequent multi-organ complications, and long-term neurologic sequelae remain limited. Expanding the literature in this area is essential for improving early recognition and optimizing acute management strategies. This report describes a case of a 16-year-old girl who developed seizures and cardiac arrest following an estimated ingestion of 4.2 g of extended-release bupropion (90.3 mg/kg), highlighting the clinical challenges in managing severe bupropion toxicity.

## 2. Case Description

A 16-year-old girl (body weight 46.5 kg) with a history of major depressive disorder and suspected bipolar affective disorder, treated with lithium 300 mg daily, bupropion (Wellbutrin XL) 150 mg daily, and fluoxetine 20 mg daily, was brought to the emergency department following an out-of-hospital cardiac arrest (OHCA). Earlier that day, she reported general discomfort and stayed home from school. By noon, she developed increasing agitation and progressive altered consciousness, eventually collapsing with generalized limb stiffness. Her mother initially misinterpreted the event as a temper tantrum. At that time, her behavior appeared unusual but not immediately alarming to her family, which contributed to a delay in recognizing the severity of her neurological deterioration.

At approximately 3:16 p.m., the patient became cyanotic and unresponsive and displayed persistent limb rigidity, prompting her mother to call emergency medical services (EMS). Upon EMS arrival, she was found seizing in the prone position, with perioral cyanosis and continuous convulsions. After repositioning, EMS noted pulselessness and initiated cardiopulmonary resuscitation (CPR). The initial cardiac rhythm was asystole. Return of spontaneous circulation (ROSC) was achieved after approximately 21 min of CPR, and the patient was transported to the hospital. Epinephrine was administered according to standard advanced life support (ALS) protocols in the prehospital setting, and endotracheal intubation was performed in the emergency room. The prolonged resuscitation effort highlights the severity of her presentation, as extended low-flow states are strongly associated with significant systemic and neurologic compromise.

**Figure 1:**
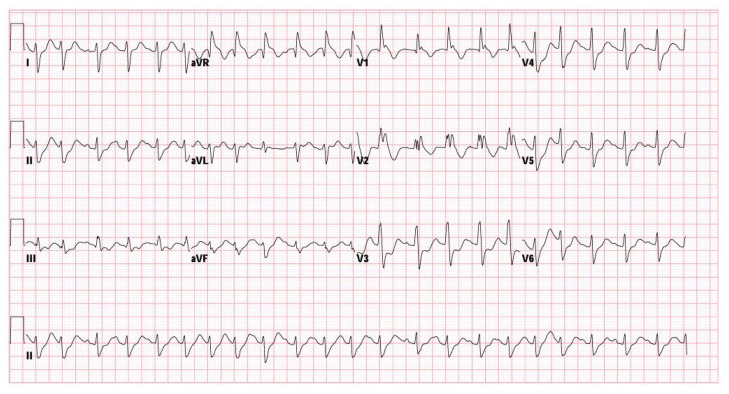
Initial ECG showing right bundle branch block and prolonged QTc interval.

On arrival at the emergency department, her vital signs showed hypotension (blood pressure 85/50 mmHg) and tachycardia (heart rate 110 bpm). Neurologically, she was comatose (GCS: E1VEM1) with bilaterally dilated, fixed pupils (5 mm). Laboratory evaluation revealed severe metabolic acidosis (pH 6.648, bicarbonate 6.4 mmol/L, lactate 20.0 mmol/L), hyperglycemia (glucose 247 mg/dL), and leukocytosis (white blood cell count 22,000/μL) (Table 1). Sodium bicarbonate (40 mL IV, 70 mg/mL) was given to correct the severe metabolic acidosis noted shortly after ROSC. Electrocardiography (ECG) showed a new right bundle branch block (QRS interval 132 ms) and a prolonged QT interval (QTc 510 ms) (Figure 1). A urine toxicology screen was negative for most recreational drugs, except for benzodiazepines, consistent with her prescribed medications. These findings collectively indicated profound physiological stress and suggested that both severe hypoxia and toxicologic factors contributed to her critical state. Following initial resuscitation and stabilization, the patient was admitted to the pediatric intensive care unit (PICU) on the same day.

**Figure 2:**
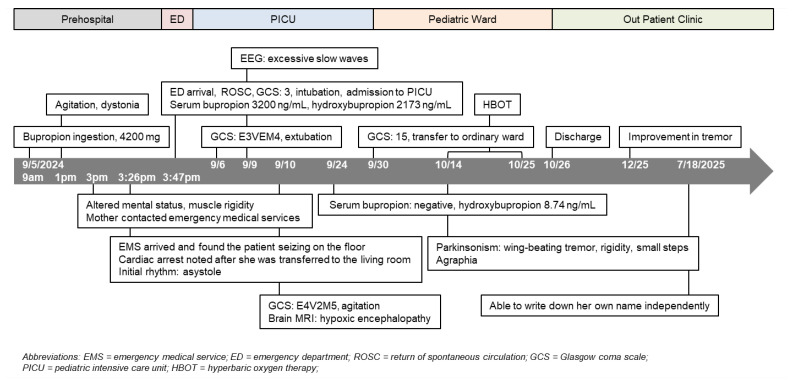
Clinical timeline.

A review of the patient’s medications revealed that approximately one month’s supply of bupropion (estimated 28 tablets, totaling 4200 mg or 90.3 mg/kg) was missing. Her family suspected the ingestion occurred around 9 to 10 a.m., approximately 6 to 7 h before the cardiac arrest. Serum analysis 6 h post-ingestion revealed markedly elevated concentrations of bupropion (3200 ng/mL; therapeutic range 150–250 ng/mL) and hydroxybupropion. Additionally, her clinical timeline (Figure 2) suggested that the progressive agitation observed throughout the morning might be a prodromal phase described in severe bupropion toxicity, during which early neurological excitation precedes tonic–clonic seizures. The delayed timing of collapse is also compatible with extended-release bupropion ingestion, which is known to have a prolonged absorption window and risk of delayed neurotoxicity. This pattern of delayed deterioration further supports the pharmacokinetic profile of extended-release formulations, which can maintain rising serum concentrations long after ingestion.

**Figure 3:**
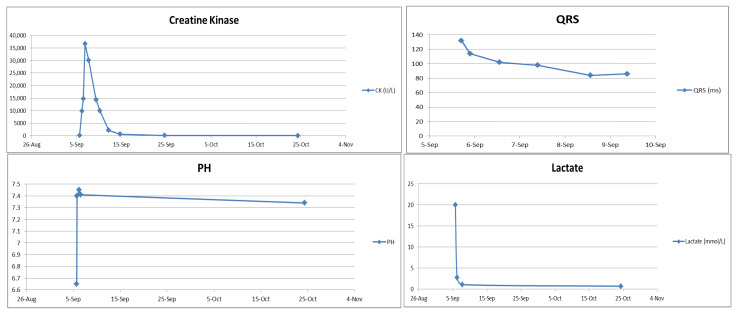
Laboratory trend (CK, QRS, pH, lactate).

**Figure 4:**
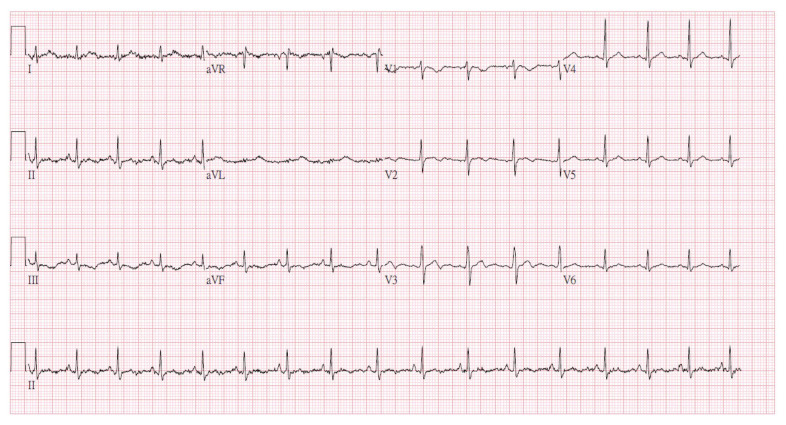
Follow-up ECG showed resolution of the prior abnormalities.

During hospitalization, the patient developed rhabdomyolysis (creatine kinase [CK] peaking at 36,636 U/L on the second day of admission) despite an initially normal CK level. The CK level dropped to 161 U/L 20 days after admission (Figure 3). Regarding medical management, intravenous levetiracetam 1000 mg every 12 h was initiated for seizure control following PICU admission. Adequate intravenous fluid hydration was provided for the treatment of rhabdomyolysis. As the patient became more responsive, significant agitation emerged; therefore, oral quetiapine 25 mg at bedtime and lorazepam 0.5 mg every 6 h were prescribed after psychiatric consultation. She was also diagnosed with hypoxic encephalopathy, later confirmed by brain MRI, and subsequently manifested Parkinsonism (including bradykinesia, rigidity, and resting tremor) and agraphia, which were identified by the attending pediatric neurologist. A lumbar puncture was also performed to exclude central nervous system infection. An awake electroencephalogram (EEG) showing excessive slow waves suggested mild-to-moderate diffuse cortical dysfunction. Cognitive assessments revealed impairments in working and short-term memory, left-sided visuospatial deficits, and calculation difficulties, consistent with hypoxic brain injury. During her stay in the pediatric ward, a comprehensive rehabilitation program—including swallowing therapy, muscle strengthening, postural control training, and endurance training—was implemented to support functional recovery. She also received hyperbaric oxygen therapy (HBOT) during her hospitalization. HBOT was initiated due to prolonged hypoxia during resuscitation and its potential neuroprotective effects in hypoxic brain injury. It was not intended as a therapeutic intervention for bupropion toxicity. Follow-up ECG on 9 September (Figure 4) showed resolution of the prior abnormalities, and serum bupropion was undetectable by hospital day 20.

Given that this was a suicidal event, psychiatric consultations were obtained. The first consultation was conducted on 11 September, during which agitation and delirium were observed while the patient was in the PICU. The psychiatrist recommended management of the underlying medical condition and pharmacologic treatment, including quetiapine, haloperidol, and lorazepam. The second consultation took place on 21 October, during which the psychiatrist determined that the patient’s neurological impairment was consistent with organic brain syndrome secondary to hypoxic encephalopathy. Her suicide risk was assessed as low to moderate. Continued rehabilitation was encouraged, and medications including quetiapine and valproic acid were recommended. The psychiatrist also advised against the use of antidepressants. The patient was discharged approximately seven weeks later in stable condition, though with residual neurological deficits.

Parkinsonism and agraphia were first noted on 14 October, approximately five weeks after the ingestion. By the end of the year, a gradual improvement in Parkinsonism was documented in the outpatient rehabilitation records. Agraphia also showed mild improvement; by July 2025, the patient was able to write her own name without assistance. A psychological assessment was performed on 17 July 2025. The score of the Wechsler Adult Intelligence Scale, Fourth Edition (WAIS-IV) was 55 (PR 0.1), suggesting mild to moderate intellectual disability. The Adaptive Behavior Assessment System-II (ABAS-II), conducted by the patient’s mother, suggested moderate disability with a score of 47 (PR < 0.1). The patient demonstrates mildly reduced digital dexterity, requiring increased effort for block construction tasks and handwriting. She has difficulty completing time-limited tasks within the prescribed intervals. During the Matrix Reasoning and Visual Puzzles subtests, the patient reported that the visual stimuli appeared highly similar, making discrimination challenging. Her attentional capacity is mildly diminished; although she can follow task transitions as directed by the examiner, she is occasionally distractible, becomes momentarily absent-minded, or forgets task rules during performance. According to the patient’s mother, she is able to eat, toilet, and bathe independently in the home or other familiar environments. She can independently don and doff loose-fitting clothing. She is sometimes able to operate the washing machine to perform laundry; however, she intermittently forgets the operational steps. Medication adherence is inconsistent, and she occasionally takes medications incorrectly, necessitating maternal supervision and assistance. Of note, her neurocognitive symptoms required continued outpatient rehabilitation, highlighting the long-term consequences of prolonged hypoxic injury following toxicological cardiac arrest.

## 3. Discussion

Bupropion—particularly its extended-release formulations—is widely prescribed for managing depressive disorders. Besides its antidepressant role, bupropion is used for smoking cessation and has been employed in weight-management programs, ADHD treatment, and occasionally for compulsive eating behaviors [1,2,3]. Pharmacologically, bupropion, a norepinephrine/dopamine reuptake inhibitor (NDRI), is classified as a substituted cathinone (a β-keto amphetamine), and both the parent drug and its major active metabolite, hydroxybupropion, exert their effects primarily by inhibiting dopamine reuptake, with a lesser impact on norepinephrine reuptake [4]. It has a half-life of about 9.6–20.9 h, and its major metabolic mechanism is through CYP2D6. Major active metabolites of bupropion include hydroxybupropion, erythrohydrobupropion, and threohydrobupropion, with a half-life of 24–37 h. Typical daily dose ranges from 150 mg to 450 mg. Chronic daily doses exceeding 450 mg are known to increase the likelihood of seizures [5,6].

Although therapeutic use of bupropion is generally safe, overdose can cause potentially life-threatening complications. In addition to its sympathomimetic effects (tachycardia, elevated blood pressure, gastrointestinal disturbances, and agitation), reported adverse events include rhabdomyolysis, cholestasis, hepatocellular dysfunction, dystonia, dyskinesia, trigeminal nerve dysfunction, mania, generalized erythrodermic psoriasis, erythema multiforme, altered vestibular and sensory function, and serum sickness. The 4-h therapeutic serum concentration of bupropion is 150–200 ng/mL, with a trough level of 50–100 ng/mL. Massive ingestions can lead to seizures (occasionally progressing to status epilepticus), widening of the QRS interval, and, in some cases, QT prolongation. Life-threatening ventricular arrhythmias have been reported in severe overdose [7].

In a retrospective study of 3504 patients exposed to bupropion, 8 deaths (0.23%) were reported [8]. Another study by Mark Simon et al. reported 7 deaths among 990 patients (0.7%) with bupropion ingestions [9]. Similarly to our case, Dr. Carson Harris reported a 26-year-old male with a bupropion overdose who developed severe hypoxia and respiratory acidosis during the early phase of resuscitation, ultimately leading to his death in 1997 [10]. Most reported fatalities associated with bupropion overdose are attributed to life-threatening ventricular dysrhythmias. However, this was not observed in our patient; no ventricular tachycardia or fibrillation was documented in either the prehospital or in-hospital setting.

Severe cardiotoxicity and widening of the QRS interval are thought to result from prolonged and delayed elevations in serum bupropion levels [11]. Cardiac sodium channel blockade was proposed as the reason for the widening of the QRS but not experimentally proven. Animal studies suggest that the underlying mechanism involves impaired intracellular communication and coupling through gap junctions, which may explain why sodium bicarbonate therapy is often ineffective in these cases [12]. Further investigation is needed to clarify the role of intravenous lipid emulsion (ILE) in managing life-threatening toxicity. Current evidence-based recommendations support the use of ILE only in cases of critical bupropion overdose when conventional treatments have failed [13,14]. The optimal regimen has yet to be established [15]. ILE is not advised for bupropion poisoning that does not involve severe cardiac compromise. For patients who present soon after ingestion or have consumed a very large amount, multiple doses of activated charcoal (AC) and the addition of whole bowel irrigation (WBI) are recommended, provided that the airway can be safely protected.

Seizures are one of the most frequently reported serious adverse effects and may be delayed for up to 24 h, particularly with sustained- or extended-release formulations. Some patients experience symptoms lasting as long as 48 h. Additionally, recreational intranasal use has been associated with euphoria, hallucinations, and seizures due to rapid mucosal absorption that bypasses first-pass hepatic metabolism. The risk of seizures increases with larger ingested doses [16,17] and is further compounded by factors such as tachycardia, altered mental status, and prolonged QTc or QRS durations on electrocardiography [9,16,18,19]. A dose-dependent relationship was observed in a 69-patient case series, in which nearly all patients who ingested more than 9 g of bupropion experienced seizures [17].

It is still uncertain whether bupropion itself or its active metabolite, hydroxybupropion, is primarily responsible for triggering seizures [5,20]. Following seizure events, elevated concentrations of both compounds have been observed, yet the precise mechanism by which hydroxybupropion may induce seizures remains unresolved. When treatment is necessary, management should focus on supportive care, with cautious administration of benzodiazepines as the main therapeutic approach.

Although Parkinsonism has been reported as a potential adverse effect of bupropion, the clinical course in our patient was not consistent with a medication-induced phenomenon. Undeberg et al. described a 54-year-old female who developed bupropion-induced tremors [21], and Parkinsonism has also been listed among bupropion-associated movement disorders in a systematic review by Pitton Rissardo et al. [22]. However, in our case, these neurological symptoms persisted despite the discontinuation of bupropion. Therefore, we believe hypoxic encephalopathy was the primary etiology of both Parkinsonism and agraphia in this patient.

Rhabdomyolysis has been reported in both therapeutic and overdose scenarios. A 49-year-old male taking bupropion 150 mg twice daily for 5 months developed an elevated CK level of 18,394 U/L, which normalized after discontinuing bupropion [23]. Raluca Ungureanu et al. described severe rhabdomyolysis following ingestion of 4 g of immediate-release bupropion [24]. The mechanism behind bupropion-related rhabdomyolysis is not fully understood but is believed to involve a combination of prolonged seizure activity, excessive sympathetic stimulation, and possible direct myotoxic effects of bupropion metabolites. Early aggressive fluid resuscitation is essential to avoid acute kidney injury. In our patient, initial CK levels were normal despite prolonged seizure activity, emphasizing the need for serial monitoring rather than relying on a single measurement. Clinicians should remain vigilant for rhabdomyolysis in these patients, particularly given the potential for delayed CK elevation, as seen in this patient.

Supportive care remains the cornerstone of treatment, as no specific antidote exists. Benzodiazepines are recommended as first-line therapy for seizures, and lidocaine may be considered for conduction abnormalities. In our case, the patient was placed under continuous ECG monitoring immediately after ROSC and throughout her admission to the PICU. Although wide QRS complexes were observed, lidocaine was not administered because no ventricular dysrhythmias were detected during monitoring. In recent years, several toxicology centers have explored additional interventions, such as extracorporeal membrane oxygenation (ECMO), for refractory cardiogenic shock, though reports remain anecdotal [25]. Continuous EEG monitoring has also been proposed for patients with prolonged altered mental status, given the risk of non-convulsive seizures following bupropion overdose. Furthermore, methods of enhanced elimination, such as hemodialysis and hemoperfusion, are not beneficial because of the large volume of distribution of bupropion and high protein binding. Though Akdemir et al. [26] have reported a 23-year-old female, who took 25–30 tablets of Wellbutrin XL (bupropion hydrochloride), successfully treated by charcoal hemoperfusion. The efficacy of enhanced elimination in the context of bupropion overdose requires further investigation.

Self-harming and suicidal behaviors by medication overdose are not uncommon in clinical practice; however, intentional overdose specifically involving bupropion is relatively less frequent. The situations described in this case report tend to occur in individuals with underlying psychiatric disorders, prior suicide attempts, substance use disorders, or poor medication adherence. Patients with recent psychosocial stressors or inadequate social support are also at increased risk. Prevention relies primarily on early identification of high-risk individuals. This includes routine assessment of suicidal ideation, close follow-up after medication changes, and careful evaluation of access to potentially harmful medications. For patients identified as being at higher risk, more frequent monitoring—such as regular psychiatric visits, closer outpatient surveillance, involving family members in treatment plans, and considering limiting the quantity of medication dispensed—may help reduce the likelihood of overdose. Overall, proactive risk assessment and timely intervention remain key strategies to prevent such events.

## 4. Conclusions

This case highlights the severe and multifaceted toxicity that can result from massive extended-release bupropion ingestion in adolescents, including seizures, cardiac arrest, profound metabolic derangements, rhabdomyolysis, and long-term neurologic impairment. The patient’s clinical course illustrates the characteristic progression from early neuroexcitation to seizure-induced hypoxia and subsequent cardiac arrest, emphasizing the importance of early recognition of prodromal symptoms in high-risk ingestions. Her delayed rhabdomyolysis and persistent neurocognitive deficits further underscore the potential for prolonged multi-organ involvement even after hemodynamic stabilization. This report reinforces the need for aggressive supportive care, continuous cardiac and neurologic monitoring, and consideration of decontamination strategies when feasible. Early multidisciplinary intervention, including neurorehabilitation, is essential for optimizing outcomes. Additional research is warranted to better define predictive factors for deterioration and to clarify the roles of emerging therapies such as intravenous lipid emulsion in severe bupropion toxicity.

## Figures and Tables

**Table 1:** Initial laboratory findings.

Laboratory Findings	Value	Reference Range
White blood cell count	22.19 × 10^3^/uL	3.5–11.0
Band	0.0%	0–3
Neutrophil	52.3%	40–75
Lymphocyte	35.7%	20–45
Monocyte	7.5%	2–10
Eosinophil	2.5%	1–6
Basophil	1.5%	0–1
Atypical lymphocyte	0.5%	0–1
Hemoglobin	13.6 g/dL	12.0–16.0
Platelet	384 × 10^3^/uL	150–400
PH (arterial gas)	6.648	7.35–7.45
PCO_2_	59.2 mmHg	35–45
PO_2_	149.1 mmHg	80–100
Bicarbonate	6.4 mmol/L	22–26
Sodium	140 mmol/L	136–145
Potassium	4.0 mmol/L	3.5–5.1
Glucose	247 mg/dL	70–100
Blood urea nitrogen	13 mg/dL	7–25
Creatinine	1.08 mg/dL	0.6–1.1
Alanine aminotransferase	43 U/L	<41
Bilirubin	0.21 mg/dL	0.3–1.0
Lactate	20.0 mmol/L	0.5–2.2
Creatine kinase	124 U/L	30–223

## Data Availability

The data presented in this study are available on request from the corresponding author due to privacy or ethical restrictions.
